# Dominant optic atrophy

**DOI:** 10.1186/1750-1172-7-46

**Published:** 2012-07-09

**Authors:** Guy Lenaers, Christian Hamel, Cécile Delettre, Patrizia Amati-Bonneau, Vincent Procaccio, Dominique Bonneau, Pascal Reynier, Dan Milea

**Affiliations:** 1 Institut des Neurosciences de Montpellier, U1051 de l’INSERM, Université de Montpellier I et II, BP 74103, , F-34091, Montpellier cedex 05, France; 2 CHRU Montpellier, Centre de Référence pour les Maladies Sensorielles Génétiques, Hôpital Gui de Chauliac, F-34295, Montpellier, France; 3 INSERM U1083, F-49000, Angers, France; 4 CNRS 6214, F-49000, Angers, France; 5 Centre Hospitalier Universitaire, F-49000, Angers, France; 6 Glostrup University Hospital, Copenhagen, Denmark

## Abstract

**Definition of the disease:**

Dominant Optic Atrophy (DOA) is a neuro-ophthalmic condition characterized by a bilateral degeneration of the optic nerves, causing insidious visual loss, typically starting during the first decade of life. The disease affects primary the retinal ganglion cells (RGC) and their axons forming the optic nerve, which transfer the visual information from the photoreceptors to the lateral geniculus in the brain.

**Epidemiology:**

The prevalence of the disease varies from 1/10000 in Denmark due to a founder effect, to 1/30000 in the rest of the world.

**Clinical description:**

DOA patients usually suffer of moderate visual loss, associated with central or paracentral visual field deficits and color vision defects. The severity of the disease is highly variable, the visual acuity ranging from normal to legal blindness. The ophthalmic examination discloses on fundoscopy isolated optic disc pallor or atrophy, related to the RGC death. About 20% of DOA patients harbour extraocular multi-systemic features, including neurosensory hearing loss, or less commonly chronic progressive external ophthalmoplegia, myopathy, peripheral neuropathy, multiple sclerosis-like illness, spastic paraplegia or cataracts.

**Aetiology:**

Two genes (*OPA1*, *OPA3*) encoding inner mitochondrial membrane proteins and three loci (OPA4, OPA5, OPA8) are currently known for DOA. Additional loci and genes (OPA2, OPA6 and OPA7) are responsible for X-linked or recessive optic atrophy. All *OPA* genes yet identified encode mitochondrial proteins embedded in the inner membrane and ubiquitously expressed, as are the proteins mutated in the Leber Hereditary Optic Neuropathy. *OPA1* mutations affect mitochondrial fusion, energy metabolism, control of apoptosis, calcium clearance and maintenance of mitochondrial genome integrity. *OPA3* mutations only affect the energy metabolism and the control of apoptosis.

**Diagnosis:**

Patients are usually diagnosed during their early childhood, because of bilateral, mild, otherwise unexplained visual loss related to optic discs pallor or atrophy, and typically occurring in the context of a family history of DOA. Optical Coherence Tomography further discloses non-specific thinning of retinal nerve fiber layer, but a normal morphology of the photoreceptors layers. Abnormal visual evoked potentials and pattern ERG may also reflect the dysfunction of the RGCs and their axons. Molecular diagnosis is provided by the identification of a mutation in the *OPA1* gene (75% of DOA patients) or in the *OPA3* gene (1% of patients).

**Prognosis:**

Visual loss in DOA may progress during puberty until adulthood, with very slow subsequent chronic progression in most of the cases. On the opposite, in DOA patients with associated extra-ocular features, the visual loss may be more severe over time.

**Management:**

To date, there is no preventative or curative treatment in DOA; severely visually impaired patients may benefit from low vision aids. Genetic counseling is commonly offered and patients are advised to avoid alcohol and tobacco consumption, as well as the use of medications that may interfere with mitochondrial metabolism. Gene and pharmacological therapies for DOA are currently under investigation.

## Review

### **Disease name/synonyms**

DOA: Dominant Optic Atrophy (OMIM #165500), initially called Kjer’s Optic Atrophy, was first described by the Danish ophthalmologist Dr. Poul Kjer [1]. DOA is also called Autosomal Dominant Optic Atrophy (ADOA), to emphasize its autosomal mode of inheritance, in contrast with Leber Hereditary Optic Neuropathy (LHON), inherited by mutations on the mitochondrial genome and maternal lineage.

DOAD-DOA*plus*: Dominant Optic Atrophy and Deafness and DOA*plus* (both OMIM #125250) are syndromic forms of DOA associating neurosensory deafness (DOAD) and/or other clinical manifestations (DOA*plus*) like myopathy, progressive external ophthalmoplegia, peripheral neuropathy, stroke, multiple sclerosis or spastic paraplegia.

DOAC: Dominant Optic Atrophy and Cataract (OMIM #606580) is a rare form of DOA associated to cataract.

Orphanet reference numbers are ORPHA98673 for DOA, and ORPHA1215 for DOA*plus*.

### Definition

DOA is an optic neuropathy due to the degeneration of optic nerve fibers. It belongs to the group of inherited optic neuropathies (ION), which are genetic conditions affecting the retinal ganglion cells (RGCs) whose axons form the optic nerve. Because RGCs are neurons originating from an extension of the diencephalon, DOA is a disease of the central nervous system [2].

DOA is a mitochondriopathy, as the genes responsible for DOA encode proteins ubiquitously expressed, imported into the mitochondria and associated to the inner membrane [3]. As such, DOA may be syndromic and include extra-ocular symptoms, mostly neuro-muscular, that are frequently found in mitochondriopathies [4].

### Epidemiology

DOA is a relatively common form of inherited optic neuropathy. Its prevalence is 3/100,000 in most populations in the world, but can reach 1/10,000 in Denmark where a founder effect was identified [5, 6]. DOA penetrance is around 70%, but depending on families, mutations and study criteria [6,7], it can vary from 100% [5] to 43% [8]. Syndromic DOAD and DOA*plus* account for some 20% of all DOA cases and are fully penetrant [9].

### Clinical description

The disease was first described at the end of the 19^th^ century [10,11]. Large families were then reported in UK [12], USA [13] and France [14], but it was after the report of 19 DOA families by the Danish ophthalmologist Kjer that this clinical entity was recognized and assigned his name [1].

#### Non syndromic dominant optic atrophy

In most cases, DOA presents as a non syndromic, bilateral optic neuropathy. Although DOA is usually diagnosed in school-aged children complaining of reading problems, the condition can manifest later, during adult life [15-17]. DOA patients typically experience a slowly progressive, insidious decrease of vision, which can rarely be asymmetric, although rapid decline has also been reported in adults [18,19]. The visual impairment is irreversible, usually moderate (visual acuity: 6/10 to 2/10) and highly variable between and within families. However, extreme severity (legal blindness) or very mild presentation (subclinical decrease in visual acuity) can be encountered [20,21].

**Figure 1:**
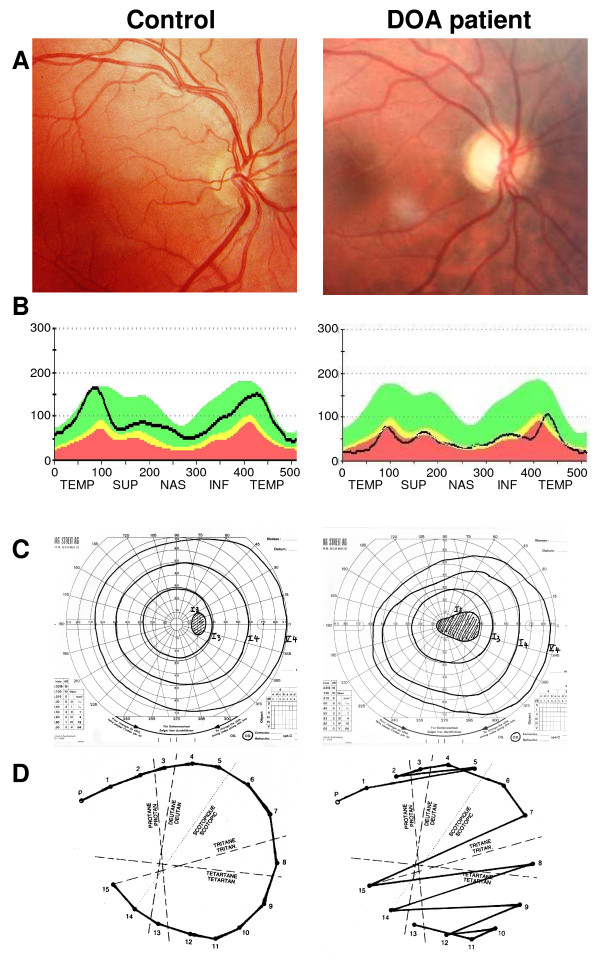
**Ophthalmological description of a DOA patient.** Results from ophthalmological examination of a paradigm Dominant Optic Atrophy patient with the c.2708delTTAG mutation in *OPA1* (Right) compared to a control patient (Left). (**A**): Eye fundus examination showing the pallor of the optic nerve in the DOA patient, in particular on the temporal side, whereas the rest of the retina appears totally unaffected. (**B**): Optical Coherence Tomography measures of the retinal nerve fiber layer thickness (black line), at the emergence of the optic disc. In a DOA patient, there is a general reduction in all quadrants, prevailing on the temporal side, compared to a control patient. (**C**): Visual field examination disclosing the caeco-central scotoma in the DOA patient, whereas only the blind spot is detected in control patient. (**D**): Results from a desaturated 15-Hue test presenting the characteristic tritanopia (blue-yellow axis) dyschromatopsia defect in the DOA patient.

On fundus examination, the optic disk typically presents a bilateral and symmetrical pallor of its temporal side, witnessing the loss of RGC fibers entering the optic nerve (Figure 1A). The optic nerve rim is atrophic and a temporal grey crescent is often present. Optic disc excavation is not unusual, but its clinical features vary in most of the cases from that of glaucoma. Optical Coherence Tomography (OCT) discloses the reduction of the thickness of the peripapillary retinal nerve fiber layer in all four quadrants, but does not disclose alteration of other retinal layers [22,23] (Figure 1B). The visual field typically shows a caecocentral scotoma, and less frequently a central or paracentral scotoma, while peripheral visual field remains normal (Figure 1C). Importantly, there is a specific tritanopia, i.e. a blue-yellow axis of color confusion, which, when found, is strongly indicative of Kjer disease [24,25] (Figure 1D). However, in severe cases or in patients with congenital dyschromatopsia (daltonism), interpretation of the color vision defect may be more difficult. The pupillary reflex and circadian rhythms are not affected, suggesting that the melanopsin RGC are spared during the course of the disease [26,27].

Some patients harboring the pathogenic *OPA1* mutation can be asymptomatic; at the opposite end of the clinical variability spectrum, mutations of the *OPA1* gene have been reported to enhance multisystemic deficits while sparing totally the optic nerve.

Anterior and/or posterior blue-dot cerulean cataract occurs in the rare DOA patients with an *OPA3* mutation [28].

Although typical DOA is associated with a progressive and irreversible loss of vision, we reported the case of a young man (23 years) who developed an isolated, progressive, painless bilateral optic neuropathy as a result of central scotomas that spontaneously recovered partial vision six months later. The patient harbored a novel heterozygous mutation in *OPA1* exon 5b (c.740G > A) which was the first mutation to be described in one of the three alternative *OPA1* exons [29]. In addition, we identified another original case presenting a late onset (62 years) sequencial and acute loss of vision, associated to a novel dominant mutation (c.2794C > T) in *OPA1*[30], suggesting that atypical natural histories of DOA can be related to *OPA1* mutations.

#### Syndromic dominant optic atrophy

Syndromic DOAD and DOA*plus* patients experience full penetrance and usually more severe visual deficits [9,31,32].

DOAD and DOA*plus* with extra-ophthalmological abnormalities represent up to 20% of DOA patients with an *OPA1* mutation [6]. The most common extra-ocular sign in DOA is sensori-neural hearing loss, but other associated findings may occur later during life (myopathy and peripheral neuropathy), suggesting that there is a continuum of clinical presentations ranging from a mild “pure DOA” affecting only the optic nerve, to a severe and multisystemic presentations. Sensori-neural hearing loss associated to DOA may range from severe and congenital to subclinical [31-36] with intra- and inter- familial variations, and mostly segregate with the *OPA1* R445H (c.1334G>A) mutation. In general, auditory brain stem responses, which reflect the integrity of the auditory pathway from the auditory nerve to the inferior colliculus, are absent, but both ears show normal evoked oto-acoustic emissions, reflecting the functionality of presynaptic elements and in particular that of the outer hair cells [37].

Peripheral axonal sensory and/or motor neuropathy and proximal myopathy may be diagnosed in some DOA patients from their third decade of life onwards, as well as a combination of cerebellar and sensory ataxia in adulthood, multiple sclerosis-like illness and spastic paraplegia [9,16,38,39]. Progressive external ophthalmoplegia is also frequently diagnosed in syndromic DOA*plus* patients [9]. One report of a Behr syndrome associating DOA to pyramidal signs, ataxia and mental retardation was linked to an *OPA1* mutation [40] and another report describing a severe neuromuscular phenotype associated to optic atrophy was described in two *OPA1* compound heterozygote siblings [41]. Muscle biopsy from DOA*plus* patient revealed features typical of mitochondrial myopathy, as approximately 5% of all fibers were deficient in histochemical COX activity and several fibers showed evidence of subsarcolemmal accumulation of abnormal mitochondria, a phenotype known as ragged red fibers [9,31,32].

### Etiology

#### Loci and genes

DOA is not genetically highly heterogeneous, in comparison with many other ophthalmologic or neurodegenerative disorders (Table 1). The first DOA locus, OPA1, localised on 3q28 was initially considered as unique [42,43]. But since the discovery of the *OPA1* gene in 2000 [44,45], two other loci, OPA4 and OPA5, were further identified in few families (1 for OPA4 and 2 for OPA5) presenting pure DOA [46,47]. Additional loci and genes were identified as responsible for Optic Atrophy, but either with a X-linked mode of inheritance (OPA2) [48,49], a recessive mode of inheritance (OPA6 and OPA7) [50,51] or as syndromic recessive or dominant forms (OPA3 and OPA8) [28,52,53]. Thus to date, *OPA1* is the major gene responsible for DOA, accounting for at least 75% of all the patients, whereas all the other genes or loci only contribute each for less than 1% of the patient cohort [7].

**Table 1:** DOA loci and genes

**Locus**	**Chromosome**	**Gene**	**Mode of inheritance**
OPA1	3q28-29	*OPA1*	dom.
OPA2	Xp11.4-p11.21	?	X-link
OPA3	19q13.2-q13.3	*OPA3*	dom./ress.
OPA4	18q12.2-q12.3	?	dom.
OPA5	22q12.1-q13.1	?	dom.
OPA6	8q21-q22	?	ress.
OPA7	11q14.1-q21	*TMEM126A*	ress.
OPA8	16q21-q22	?	dom.

legend: dom: dominant; ress: recessive.

#### Mutations in *OPA* genes and their consequences on the mitochondrial physiology

Three genes have been identified to date, *OPA1*, *OPA3* and *TMEM126A* (*OPA7*) (Table 1); all encode mitochondrial proteins ubiquitously expressed and associated to the inner mitochondrial membrane, due to the presence of at least one transmembrane domain in their sequence [51,54,55]. In *OPA1*, 27% of the mutations are missense, 27% are splice variant, 23.5% lead to frame shift, 16.5% are nonsense and 6% are deletion or duplication [7]. Most of them are leading to a haplo-insufficiency situation where the mutated transcript is degraded by mRNA decay, thus leading to a reduction of 50% in the amount of OPA1 protein. As a direct consequence, the different mutations in *OPA1* are not related to the severity of the disease, and genotype/phenotype correlations are difficult to infer [25]. In this respect, secondary nuclear genes, but not the mitochondrial genome, are suspected to control the severity of the disease in non-syndromic patients [56]. Conversely, few missense mutations in the GTPase domain of OPA1 are responsible for syndromic cases with severe dominant negative effects [9], because the mutated protein might interfere with and inhibit the wild-type protein. Importantly, sporadic cases, cases with *de novo* mutation and cases with unknown familial history, account together for 50% of all patients. Concerning *OPA3*, indirect evidences suggest that the 2 mutations so far reported in DOAC affect the trans-membrane domain, are fully penetrant and act in a dominant negative manner, as heterozygous carriers of a recessive mutation leading to the inhibition of *OPA3* expression are asymptomatic [52]. In the case of *TMEM126a*, the consanguineous recessive disease is associated to a mutation introducing a stop codon at position 55, thus deleting 140 out of the 195 amino-acids composing the protein [51].

Analysis of OPA1 functions in common cell lines (HeLa, COS) and dysfunctions in patient fibroblasts revealed a systematic susceptibility to apoptosis and mild to severe alteration of the mitochondrial respiration activity, essentially associated to a reduced energetic coupling [28,51,57-60]. In addition, the 8 OPA1 isoforms that result from alternate splicing of 3 exons (4, 4b and 5b) have discrete functions in structuring the cristae, in mitochondrial membrane dynamics, maintenance of the membrane potential, calcium clearance, interaction with the respiratory chain complexes and maintenance of mitochondrial genome integrity [61-65]. As a consequence, and as revealed by numerous patient fibroblast studies, mutations in *OPA1* can have a direct although variable impact on these functions, [31,33,57-59,66], and possibly the genetic background and aging might contribute to the mitochondrial phenotype, either in a compensatory or in an accentuating manner.

Importantly, the *OPA1* gene is the fifth identified nuclear gene responsible for generating multiple deletions in the mitochondrial DNA, together with *POLG1* (DNA polymerase γ), *PEO1* (twinkle), *SLC25A4* (ANT1) and *TP* (thymidine phosphorylase). The presence of multiple deletions in the mtDNA has been found in the skeletal muscle of the majority of patients harbouring OPA1 mutations, even in those with isolated optic atrophy [67]. This OPA1 related genomic instability is likely to play a crucial role in the pathophysiology of DOA, taken into account its direct functional consequence on respiratory chain capacities and may explain the convergence of clinical expressions between DOA*plus* syndromes and other disorders related to mutations in mtDNA.

#### Optic nerve and animal models

The major concern in studying DOA pathophysiology concerns the question why RGCs are most specifically affected by this disease, while the *OPA* genes are expressed in all cells of the body. Histochemical studies revealed a peculiar distribution of mitochondria in retinal ganglion cells. Indeed, they are accumulated in the cell bodies and in the intra-retinal unmyelinated axons, where they form varicosities, and are conversely scarce in the myelinated part of axons after the lamina cribosa [68-71]. These observations emphasize the importance of mitochondrial network dynamic in order to maintain the appropriate intracellular distribution of the mitochondria that is critical for axonal and synaptic functions, and point to a possible pathophysiological mechanism associated to OPA1 that could jeopardize RGC survival. Alternatively, RGCs are the only neurons of the body that are exposed to the day long stress of light, which generates oxidative species favoring the apoptotic process [72]. Therefore the mitochondrial fragility conferred by *OPA1* mutations, together with the photo-oxidative stress could precipitate RGCs in premature cell death. A third pathophysiological hypothesis involves the tremendous energetic requirements of RGCs, as these neurons permanently fire action potentials, in addition through axons that are not myelinated in the eye globe. As the energetic fuelling of RGCs soma is restricted in the central part of the retina, due to the physical constraints imposed by the macula blood vessel organization, one can hypothesize that due to the uncoupling of mitochondrial respiration in *OPA1* cells, the ATP synthesis in patient RGCs is limited and can not fulfill the physiological energetic requirements for long term cell survival. Which of these hypotheses represents the *princeps* mechanism responsible for the RGC degeneration remains unknown. Nevertheless, in the last years, two mouse models with an Opa1 mutation have been generated and deeply analyzed in terms of vision; both summarize DOA in that loss of RGCs is preeminent [73,74]. Reduction of the scotopic, but not the photopic evoked potential response was found in one mouse model [75], whereas light-adapted ERG and VEP responses revealed a significant reduction in their amplitudes in another mouse model [76]. Histological examinations revealed a decrease of the dentritic length of the RGC-On subpopulation in the retina [77], and abnormal myelin structures, increase in micro-glia and autophagy were noticed in the optic nerve [78]. In addition, some mild neuromuscular symptoms were found, as locomotor activity was reduced and tremor observed in old animals, but no alteration of the audition was detected [79], thus these Opa1 animals show some features of the syndromic DOA forms.

### Diagnostic methods

#### Anamnesis

Interviewing patients about the natural history of the disease, at best in the presence of the family, is mandatory to define the timing of visual loss over time. Suspicion of DOA prompts also the search of similar visual signs among relatives. Special attention should be paid on sensorial or peripheral neuropathy symptoms that would support the hypothesis of a pathophysiology related to a mitochondrial deficit. Finding at least one affected member in two consecutive generations is indicative of a dominant trait, or eventually of a mitochondrial maternal transmission, that will further orientate the genetic investigations.

#### Ophthalmological examination

DOA is characterized by a bilateral symmetric vision loss. On funduscopic examination, the cardinal sign consists in an optic nerve pallor usually bilateral and symmetric on the temporal side in about 50% of patients and global in the other 50% [80], especially in old or severely affected patients. In moderate cases, the optic nerve atrophy may not be visible. The neuroretinal rim is often pale and sometimes associated with a temporal pigmentary grey crescent. OCT examination discloses and quantifies the thinning of the fiber layer in the 4 cardinal directions at the optic nerve rim [23,81]. Profound papillary excavation is reported in 21% of eyes from *OPA1* patients [82]. Visual fields examination typically reveals a central, centrocecal or paracentral scotoma, which may be large in severely affected individuals, and the sparing of the peripheral visual field [20]. Color vision, evaluated by the desaturated 15-Hue test discloses often a blue-yellow loss dyschromatopsy, or tritanopia [25].

#### Electrophysiological assessment

Visual evoked potentials (VEPs) are typically absent or delayed, but are not characteristic of the disease. In subclinical or mildly affected patients, no alteration of the VEPs can be found. Pattern electroretinogram (PERG) shows an abnormal N95:P50 ratio, with reduction in the amplitude of the N95 waveform suggesting alterations of the ganglion cells layer [83].

#### Genetic investigations

The clinical diagnosis of an optic atrophy will orientate the genetic investigations. After collecting 5ml of blood of the patient and its relatives and preparing total DNA, the analysis of *OPA1* gene will be performed on the DNA sample of the index patient by amplifying and sequencing all the 31 coding exons and their flanking intronic regions. If a mutation is identified, its segregation in the family must be analyzed and its identity has to be compared to the database hosted by the CHU of Angers, France ( http://lbbma.univ-angers.fr/lbbma.php?id=9) to find out if the mutation is already recognized as pathogenic. If not, the possible consequence of the mutation on *OPA1* transcript and protein integrity should be analyzed *in silico*, and by assessing the expression of the mutated allele by RT-PCR amplification and sequencing. If no significant mutation is found in *OPA1*, then the presence of a deletion in the gene can be tested with the Multiplex Ligation Probe Amplification methodology [84-86]. Otherwise careful reconsideration of the anamnesis might orientate to test either *OPA3* gene or the full length mitochondrial genome. If results are still negative, then when the family is large and many members are affected, genetic analysis of chromosome markers can be performed to identify the causative locus and eventually a novel pathogenic gene. Nevertheless, the identification of a morbid mutation greatly helps the genetic counseling.

#### Syndromic cases

Patients with extra-ophthalmological symptoms should be referred to diagnostic centers specialized in mitochondrial disorders, in order to obtain additional examinations by a multidisciplinary team including geneticists, neuro-ophthalmologists, neurologists, otorhynolaryngologists.

The diagnosis of such multisystemic mitochondrial disorders often requires the study of the functionality of the respiratory chain in order to evaluate the severity of the energetic deficiency. A skeletal muscle biopsy is usually performed to measure the enzymatic activity of the 5 respiratory complexes and the mitochondrial oxygraphy. In addition, it allows anatomo-pathological examinations to check for the presence of mtDNA deletions, cytochrome *c* deficient fibers and ragged red fibers. Alternatively, skin fibroblasts are also useful to evaluate the severity of respiratory chain dysfunction.

### Differential diagnosis

The DOA differential diagnosis list includes all the causes of bilateral optic neuropathies, i.e. compressive, inflammatory, demyelinating, ischemic, glaucomatous, toxic, and metabolic optic neuropathies. However, an appropriate clinical and para-clinical work-up, including neuro-imaging, biochemical studies or genetic tests, will rule out these causes in most of the cases.

Among these differential diagnosis, normal tension glaucoma (NTG) may present with signs consistent with DOA, such as visual field defects and optic disc excavation. However, NTG occurs late during the adulthood and central visual loss does not occur until late during the course of the disease. Interestingly, certain allelic sequence variants in *OPA1* have been found to be more prevalent in NTG patients in comparison with controls, thus suggesting some cross-talk between the pathophysiological mechanisms of these diseases [87].

Other acquired optic neuropathies with similar presenting signs as DOA include the nutritional/toxic optic neuropathies, which may have a mitochondrial dysfunction basis. Among the toxic optic neuropathies, the most common is the tobacco-alcohol related optic neuropathy. Other possible agents causing a toxic optic neuropathy include methylene, ethylene glycol, cyanide, lead, and carbon monoxide. Finally, certain medications, including ethambutol, isoniazid, disulfiram can cause a toxic optic atrophy.

Other hereditary optic neuropathies, such as Leber’s hereditary optic neuropathy, Wolfram’s syndrome or other neuropathies associated with neurological diseases (spinocerebellar ataxias, Friedreich’s syndrome, Charcot Marie-Tooth type 2A, Deafness-Dystonia-Optic Neuropathy syndromes etc.) may, at times, present with similar signs as DOA, though the general context and the neurological signs help to differentiate those entities.

#### Differential diagnosis associated to the OPA loci

Athough OPA loci are all primarly associated to optic atrophy, in some cases they can be differentiated by the presence of secondary symptoms (Table 2) that may orient toward a particular gene or locus. *OPA2*: Two families mapping on the OPA2 locus Xp11.4-p11.21 were identified [48], both presented optic atrophy from early childhood [49], and one associated in some instances optic atrophy to mental retardation and neurological symptoms as jerks, dysarthria, dysdiadochokinesia, tremor and gait [88,89]. In both families, only male are affected and female carriers showed no abnormalities.

**Table 2:** Possible symptoms associated to optic atrophy

**Locus**	**Optic Atrophy**	**Deafness**	**Poly neuropathy**	**Multiple Sclerosis**	**Myopathy CPEO**	**Cardiopathy**	**Cataract**
OPA1	+	+/-	+\-	+\-	+\-	-	-
OPA2	+	-	-	-	-	-	-
OPA3	+	-	+\-	-	-	-	+
OPA4	+	-	-	-	-	-	-
OPA5	+	-	-	-	-	-	-
OPA6	+	-	-	-	-	-	-
OPA7	+	+\-	-	-	-	+\-	-
OPA8	+	+\-	-	-	-	+\-	-

legend: (+): systematic; (+/-): possible; (-): never reported.

*OPA3*: Patients presenting dominant mutation in *OPA3* gene display an early optic atrophy followed by a later anterior and/or posterior cortical cataract and dyschromatopsy without systematic axis. In some cases, patients present tremor, extrapyramidal rigidity, *pes cavus* and absence of deep tendon reflex [28]. Patients with *OPA3* recessive mutations present the syndromic Costeff syndrome (see next paragraph).

*OPA4* and *OPA5*: three families linked to the OPA4 or OPA5 loci present an optic atrophy that can not be differentiated from the phenotype observed in *OPA1* patients: i.e. optic nerve pallor, decreased visual acuity, color vision defects, impaired VEP, and normal ERG and no extra-ocular findings [46,47].

*OPA6* and *OPA7*: Recessive forms of optic atrophy were described linked to the OPA6 and OPA7 loci. OPA6 patients present an early onset optic atrophy slowly progressing with a red-green dyschromatopsia [50]. Concerning OPA7, a severe juvenile-onset optic atrophy with central scotoma was found in a large multiplex inbred Algerian family and subsequently in three other Maghreb families with the same mutation in the *TMEM126A* gene, suggesting a founder effect. In these family, some patients presented mild auditory alterations and hypertrophic cardiopathy [51].

*OPA8*: One large family with a optic atrophy undistinguishable from that related to *OPA1* was recently described. In this family late-onset sensorineural hearing loss, increases of central conduction times at somato-sensory evoked potentials, and various cardiac abnormalities were also described in some patients [53].

#### DOA differential diagnosis with other hereditary optic neuropathies

Leber Hereditary Optic Neuropathy (LHON) is the major differential diagnosis for optic atrophy type 1 (OPA1). LHON typically presents in young adults as painless acute or subacute visual failure, occurring sequentially in both eyes, within six months. The acute phase begins with blurring of central vision and color desaturation. The central visual acuity deteriorates to the level of counting fingers in up to 80% of cases, associated with a large centrocecal scotoma. In few cases, in particular in patients with the m.14484 G > A mutation, visual acuity may partially improve over time. Males are more commonly affected than females and women tend to develop the disorder slightly later in life and may be more severely affected, sometimes with associated multiple sclerosis like symptoms. Other neurologic abnormalities, such as a postural tremor or the loss of ankle reflexes are also found. LHON is maternally inherited by mutation in the mitochondrial genome, in most patients (95% of cases) by one of the three mutations m.11778G > A, m.14484T > C, and m.3460G > A [90].

#### Differential diagnosis between syndromic DOA and other diseases

##### *Wolfram syndrome*

Mutations in the WFS1 gene are generally associated with optic atrophy as part of the autosomal recessive Wolfram syndrome phenotype (DIDMOAD, diabetes insipidus, diabetes mellitus, optic atrophy, deafness) [91,92] or with autosomal dominant progressive low-frequency sensori-neural hearing loss that can be associated with DOA, with or without impaired glucose regulation [93,94], supporting the notion that mutations in *WFS1* as well as in *OPA1* may lead to optic atrophy combined with hearing impairment.

##### *Costeff syndrome*

Truncating mutations in *OPA3* gene are responsible for 3-methylglutaconic aciduria type 3, a recessive neuro-ophthalmologic syndrome consisting of early-onset bilateral optic atrophy and later-onset spasticity, extra-pyramidal dysfunction, and cognitive deficit. Urinary excretion of 3-methylglutaconic and 3-methylglutaric acids is increased [52,95].

##### *Charcot-Marie-Tooth type 2A2*

(CMT2A) is a peripheral distal neuropathy with optic atrophy designated as hereditary motor and sensory neuropathy type VI (HMSN VI) [96]. HMSN VI families display subacute onset of optic atrophy and subsequent slow recovery of visual acuity in 60% of affected individuals. In each pedigree a dominant mutation in the *MFN2* gene coding the outer mitochondrial dynamin mitofusin 2, was identified [97]. Recently a novel mutation in *MFN2* as been described in a patient with a DOA*plus* clinical presentation, featuring also mtDNA deletions in the calf muscle [98].

##### Deafness-dystonia-optic neuronopathy syndrome

 (DDON) is a disease associating slowly progressive decreased visual acuity from optic atrophy beginning at about 20 years of age with neuro-sensorial hearing impairment, slowly progressive dystonia or ataxia and dementia beginning at about 40 years of age. Neurologic, visual, and neuropsychiatric symptoms vary in degree of severity and rate of progression [99]. As the inheritance is X-linked, males are only affected, although females may present mild hearing impairment and focal dystonia. The DDON syndrome is linked to mutation in *TIMM8A* or to a deletion at Xq22, also causing X-linked agammaglobulinemia due to the disruption of the *BTK* gene located telomeric to *TIMM8A*[100].

##### *Other inherited disorders of Oxidative Phosphorylation*

Mitochondrial diseases featuring a defect in the respiratory chain affect about 1/4000 individuals. They include clinical presentations with widely differing genetic origin and phenotypic expression. Their clinical expression is mainly neuromuscular and neurosensorial, but the major physiological systems and functions may also be affected. More than a hundred pathogenic mutations have been described in mitochondrial DNA since 1988, and new mutations are still regularly being reported. MtDNA mutations may be secondary to the mutations of nuclear genes encoding the proteins that ensure the maintenance of mtDNA. Since 1995, more than 70 nuclear genes have been involved in respiratory chain defects. The clinical defects identified in DOA*plus* (deafness, peripheral neuropathy, chronic external ophthalmoplegia, myopathy, encephalopathy, multiple sclerosis-like syndromes) are typical of those found in multisystemic mitochondrial diseases that often themselves include optic atrophy. Thus, facing a multisystemic mitochondrial syndrome with optic atrophy it is important to check for *OPA1* mutations, but many other mitochondrial diseases not related to *OPA1* can also display a clinical presentation similar to DOA*plus,* as recently evidenced by the discovery of a singular mis-sense mutation in the *MFN2* gene leading to a DOA*plus* phenotype [98]. Interestingly, in a few cases, the clinical presentations of *OPA1* mutations excluded optic nerve involvement [9,101], suggesting that rare *OPA1*-associated diseases may tend towards clinical phenotypes far removed from the initial description of DOA.

### Genetic counseling

DOA is inherited as an autosomal dominant trait. When the causative mutation has been identified either in *OPA1* or *OPA3* genes, it should be present in one of the parents except in *de novo* cases, and will be transmitted with a 50% chance to the proband sibs. When the causative mutation is not identified, genetic analysis can be performed on the family if other members are affected, in order to localize the locus responsible for the disease. Nevertheless, in these latter cases, results might not be straightforward and genetic counseling could remain doubtful. Otherwise, when facing simplex proband without known gene etiology, no genetic counseling can be provided. Importantly, in isolated cases, *de novo* mutations were frequently reported in *OPA1* gene, allowing consequently to provide advises for family projections. In this respect, prenatal diagnosis for pregnancies at risk is feasible but remains complicated when considering the incomplete penetrance and the markedly variable inter- and intra-familial expressivity of DOA.

### Antenatal diagnosis

The optimal time for determining genetic risk and sensitizing future parents to genetic testing is obviously before pregnancy. If young adults are affected or at risk, it will be appropriate to discuss the potential risk for their offspring and the reproductive options, as pre-implantation genetic diagnosis is available for families in which the disease-causing mutation or locus has been identified. Alternatively, prenatal genetic diagnosis for pregnancies at risk is possible by analysis of DNA extracted from fetal cells obtained by amniocentesis, again when the disease-causing allele in the affected family member has been identified.

Prenatal testing for DOA is controversial and uncommon, especially since the disease does not affect intellectual development or life span. Prenatal testing implies a thorough discussion between the health care professionals and the involved parents.

### Management including treatment

The management of DOA patient consists in regular ophthalmologic examination, including measurement of visual acuity, color vision, visual fields and OCT. To date, no specific treatment exists, but low-vision aids in patients with severely decreased visual acuity can be beneficial. Avoiding tobacco and alcohol intake as well as medications (antibiotics, antivirals) which can interfere with mitochondrial metabolism can be additional prophylactic measures.

Cochlear implants have been shown to restore a marked improved audition in patients with syndromic DOA with neurosensorial deafness [37].

### Prognosis

In most cases, the diagnosis of DOA is established before adulthood. Subsequent visual loss is mild, but can at times worsen acutely, while spontaneous improvement is exceptionally rare. However, patients may develop adaptative strategies, allowing them to fixate within intact retinal regions and thus comply with a normal familial and social life, although insertion in the professional life might be compromised by the visual defect.

Patients with syndromic DOA will experience a more severe visual defect that often will be followed by audition impairment, which together will affect their social communication early in adulthood. Additional symptoms can occur later during the third or fourth decade of life, and are believed to progress slowly.

### Unresolved questions and conclusions

Although *OPA1*, the major gene responsible for DOA has now been discovered more than ten years ago, much remains to be understood to explain the specificity of the disease that focus first on the optic nerve integrity. Indeed two major challenges are unanswered: the identification of the *princeps* mechanism that is affected in DOA, and deciphering why mainly RGCs are degenerating in this disease. Answering both of these questions should facilitate the future design of treatments.

The current absence of treatment for DOA raises a tremendous challenge in testing therapeutic strategies on the different available models, from cell lines to animals. It is probably not beyond reasonable hope to think that in the next ten years, treatments will be found to restrain the RGCs loss in DOA.

## Abbreviations

DOA: Dominant Optic Atrophy; DOAD: Dominant Optic Atrophy and Deafness; DOA*plus*: syndromic Dominant Optic Atrophy; OPA: Optic Atrophy; LHON: Leber Hereditary Optic Neuropathy; ION: Inherited Optic Neuropathy; NTG: Normal Tension Glaucoma; DDON: Deafness Dystonia and Optic Neuropathy; DIDMOAD: Diabetes Insipidus, Diabetes Mellitus, Optic Atrophy and Deafness; CMT2A: Charcot Marie Tooth type 2A disease; HMSN: Hereditary Motor and Sensory Neuropathy; RGC: Retinal Ganglion Cell; VEP: Visual Evoked Potential; PERG: Pattern ElectroRetinoGram; OCT: Optical Coherence Tomography.

## Competing interests

All authors declare that they have no competing interests.

## Authors’ contributions

All authors have contributed to the redaction and correction of the manuscript. All authors read and approved the final manuscript.
